# Non-Destructive Characterization of Drywall Moisture Content Using Terahertz Time-Domain Spectroscopy

**DOI:** 10.3390/s25175576

**Published:** 2025-09-06

**Authors:** Habeeb Foluso Adeagbo, Binbin Yang

**Affiliations:** Department of Electrical and Computer Engineering, North Carolina A&T State University, Greensboro, NC 27411, USA; hfadeagbo@aggies.ncat.edu

**Keywords:** Terahertz Time-Domain Spectroscopy, non-destructive testing, dielectric properties, refractive index, permittivity, dissipation factor, absorption and extinction coefficients, material characterization

## Abstract

Despite its wide acceptance, one of the most critical limitations of Terahertz wave technology is its high sensitivity to moisture. This limitation can, in turn, be exploited for use in moisture detection applications. This work presents a quantitative, non-invasive characterization of moisture content in standard gypsum drywall using Terahertz Time-Domain Spectroscopy (THz-TDS). With an increase in the moisture content of the drywall sample, experimental results indicated an increase in the dielectric properties such as the refractive index, permittivity, absorption coefficient, extinction coefficient, and dissipation factor. The demonstrated sensitivity to moisture establishes THz-TDS as a powerful tool for structural monitoring, hidden defect detection, and electromagnetic modeling of real-world building environments. Beyond material diagnostics, these findings have broader implications for THz indoor propagation studies, especially for emerging sub-THz and low THz communication technologies in 5G/6G and THz imaging of objects hidden behind the wall.

## 1. Introduction

Terahertz Time-Domain Spectroscopy (THz-TDS) has emerged as a transformative technique in non-destructive testing (NDT) and sensing of moisture content in porous materials such as leaves, grains, wood, food items, and pharmaceuticals [1,2,3,4,5,6]. Terahertz Time-Domain Spectroscopy is a technology that operates in the submillimeter frequencies (0.1–10 THz). At this frequency range, THz waves are very sensitive to water content due to liquid water’s high dielectric dispersion and absorption properties [7]. These unique properties, combined with its lack of health risks due to its non-ionizing properties, make THz radiation a suitable technique for both indoor and outdoor in situ diagnostics, including moisture detection [5].

Over the years, THz-TDS systems have gained popularity for water content detection across diverse biological, agricultural, forestry, construction, polymer, and several other materials. Reference [1] studies water diffusion in natural cork enclosures, Refs. [3,4] illustrates moisture detection in wood, Ref. [5] estimates the water content in different plant leaves, Ref. [8] monitors the water status of plant for drought stress study, Ref. [9] demonstrates moisture detection in a recalcitrant fruit, and [10] uses THz spectroscopy to determine the effect of moisture on the dielectric properties of soil. In recent times, THz-TDS technology and other NDT techniques have been combined with machine learning algorithms for more accurate prediction of the moisture content in different materials [11,12]. These studies, which analyze changes in dielectric properties such as the refractive index, absorption coefficients, and relative permittivity, have confirmed that the contrast between the dielectric constant of water and dry materials, which in turn causes shifts in time delay, phase difference, and signal attenuation, can be used to map moisture distributions and diffusion behaviors in moist materials.

Drywall, also known as gypsum board, is one of the most widely accepted cost-effective construction materials for walls and ceilings because of its ease of installation, thermal insulation, and sound attenuation [13]. However, due to its hygroscopic nature, drywall is highly vulnerable to moisture absorption, which compromises its structural integrity. Detecting and quantifying drywall moisture content is essential for ensuring buildings’ safety, longevity, and energy efficiency. Although there are popular traditional moisture assessment techniques, such as the Karl Fischer Moisture Meter Method [14], infrared thermography [15], and nuclear methods [16], they often rely on invasive, point-based, or low-sensitivity approaches that fail to characterize internal water distribution, especially when the material is multilayered, like in the gypsum drywall.

This work presents a controlled, non-destructive methodology for analyzing the effect of moisture content on the properties of a quarter-inch-thick standard gypsum drywall in the THz frequency range 0.2–0.65 THz. The sample was first soaked in water until it was saturated, and then it as subjected to stepwise desorption through brief microwave exposures followed by ambient rest periods. The controlled desorption is aimed at simulating moisture loss without inducing structural damage. At each moisture level, the mass was recorded to quantify moisture content gravimetrically. Subsequent THz-TDS measurements were performed in the transmission mode to extract frequency-dependent dielectric properties, including the refractive index, absorption coefficient, relative permittivity, and loss tangents.

Beyond characterizing material properties, this work has broader implications for future THz-based communication systems and through-the-wall imaging technologies. With the advancement of 6G wireless technologies, which leverage the THz frequency range, understanding the behavior of THz waves in common construction materials like drywall under different environmental conditions, such as moisture, is important for realistic electromagnetic propagation modeling [17,18]. Additionally, the sensitivity of THz signals to subsurface moisture offers potential applications in security screening, non-invasive infrastructure inspection, and smart building diagnostics.

## 2. Materials and Methods

### 2.1. Drywall Sample Preparation and Moisture Conditioning

**Figure 1:**
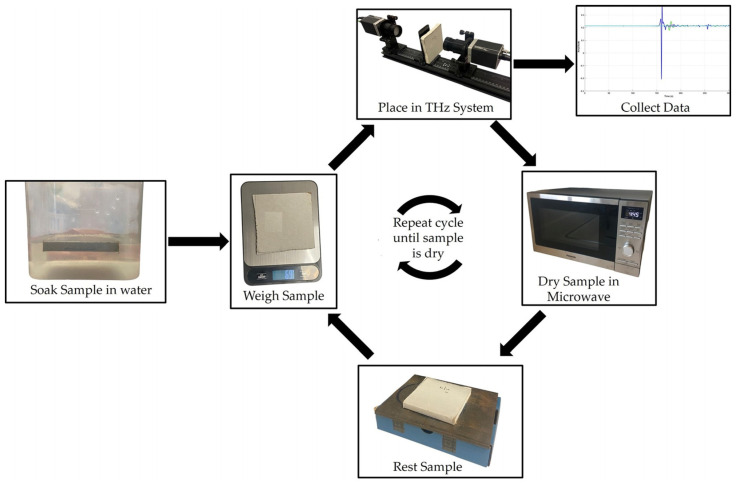
Overview of the experimental workflow.

The material under study was a standard 1/4 inch gypsum drywall, cut into a square measuring 10 cm by 10 cm. The sample retained its multilayer composition, including the gypsum core enclosed between two thin outer paper insulation layers on both sides, representing a typical construction-grade drywall segment, as shown in Figure 1.

The sample was fully immersed in water for approximately 5 min to establish the controlled moisture conditions, allowing sufficient absorption into the porous gypsum matrix. After saturation, the surface was gently dried using paper towels to remove excess surface water, and the resulting mass was measured using a 1 g/1 oz resolution kitchen scale. Moisture content was progressively reduced through a stepwise desorption approach, involving a 15-s microwave exposure followed by a 2-min rest period at ambient conditions. This cycle was repeated up to 10 times, simulating realistic drying while avoiding thermal shock and structural alterations.

The sample was weighed after each drying and resting stage to monitor the water loss. The moisture content (*WC*) was then calculated using the gravimetric-like expression normalized to the initial wet mass:

(1) $$WC=\frac{W_{stage}-W_{dry}}{W_{stage}}$$

where $W_{stage}$ is the mass at a given stage, and $W_{dry}$ is the mass of the dry sample. This controlled method enables the characterization of the same sample at multiple water content levels, ensuring consistency across all terahertz measurements.

### 2.2. Terahertz Time-Domain Spectroscopy Setup

**Figure 2:**
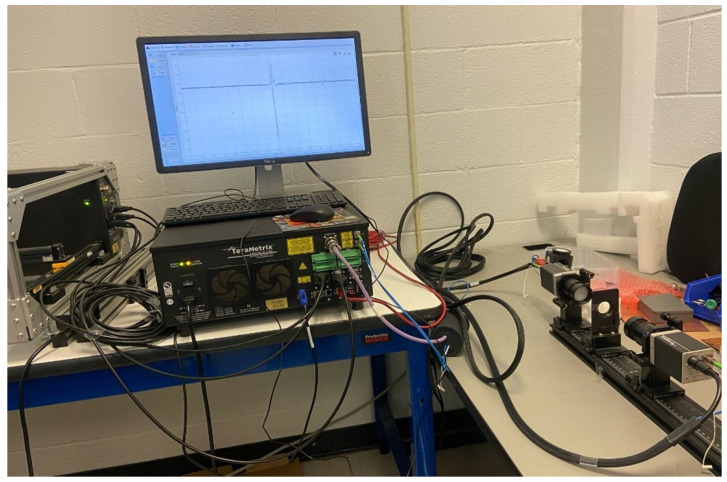
TeraMetrix T-Ray^®^ 5000 system with the emitter and detector mounted on a custom-built sample holder.

Terahertz measurements were carried out using a TeraMetrix T-Ray^®^ 5000 THz-TDS System by Luna Innovations Inc., Ann Arbor, MI, USA, Ref. [19] operating in the transmission mode as shown in Figure 2. The system covers a spectral range from 0.1 to 5 THz. It utilizes a femtosecond laser source to drive fiber-coupled photoconductive antenna-based emitter and detector modules, connected to the control unit via polarization-maintaining (PM) fiber cables. The generated THz beam is collimated and focused using a set of precision lenses attached to the antenna modules. Temporal waveforms can be captured by scanning a mechanical delay stage with picosecond resolution.

The drywall sample was mounted vertically between the emitter (transmitter) and the detector (receiver) using a custom-built sample holder shown in Figure 1, designed to minimize beam clipping and angular misalignment. Before the measurement, the system was calibrated to ensure optimal power levels, a consistent beam path, and correct polarization alignment.

At the start of the experiment, a single reference signal was acquired without any samples in the beam path. This reference signal was used as the baseline for comparison with the sample signal recorded at each moisture condition, with the drywall sample placed in the beam path. Using a fixed reference, this approach allowed for consistent comparison across all drying stages. THz measurements for the drywall sample were taken at six moisture conditions: 21.88%, 18.03%, 12.28%, 9.09%, 3.85%, and 0%. Signal acquisitions and system control were managed using the T-Ray^®^ Server Software Version 2.10.4 [20]. To ensure that the moisture content is uniformly distributed during the desorption process, at every stage of the experiment, we performed the THz-TDS measurements at three different spatially separated points on the drywall sample. The majority of the extracted TD and FD waveforms and the dielectric properties calculated from the measured waveforms at the three sample points are consistent, indicating that the moisture was evenly distributed across the drywall sample. This ensured that high-fidelity signals and accurate characterization of moisture-induced variations in the THz response were obtained throughout the experiment.

### 2.3. Time- and Frequency Domain Signal Comparison

A comparison analysis of the raw time-domain waveforms and the corresponding power spectra was conducted to evaluate the impact of moisture on terahertz wave propagation before material parameters extraction.

**Figure 3:**
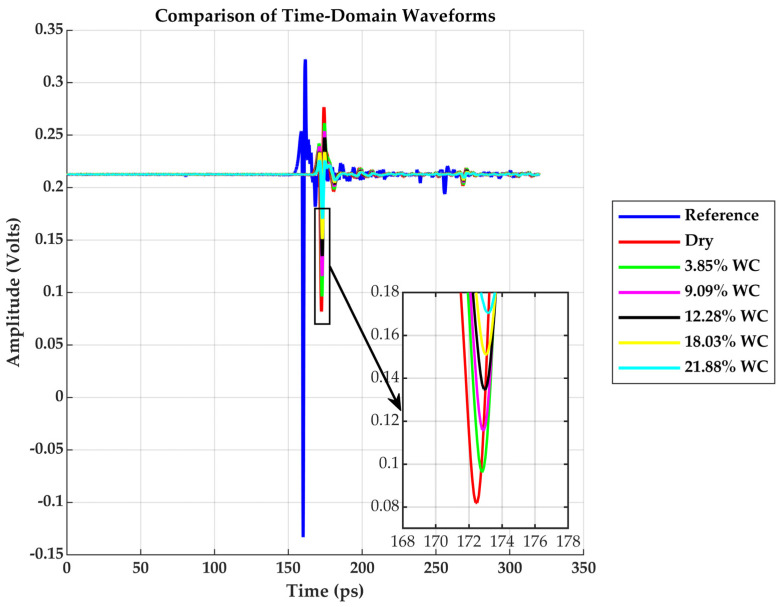
Reference and Drywall Samples Time-Domain Waveforms at different *WC*.

Figure 3 shows the temporal electric field waveforms acquired for the reference and drywall samples with varying moisture contents ranging from 0% to 21.88%. The reference waveform (blue) exhibits the strongest peak amplitudes around 0.35 V and the earliest arrival time, as expected due to negligible attenuation and delay in air. As the moisture content increases, the THz pulse undergoes amplitude attenuation and temporal delay for the drywall samples. The dry sample has a peak around 0.27 V, and the most saturated sample (21.88% *WC*) shows the lowest peak and is delayed by approximately 5 ps relative to the dry sample.

**Figure 4:**
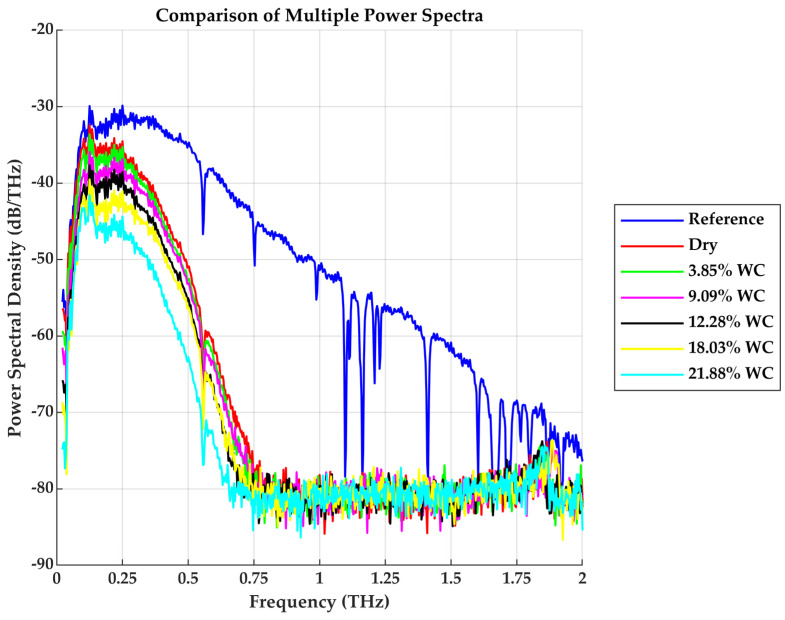
Reference and Drywall Samples Power Spectra at different *WC*.

Figure 4 presents each measurement’s corresponding power spectral density (PSD) plots. The reference shows a broad usable bandwidth extending to approximately 2 THz with peak power density around −30 dB/THz near 0.3 THz. The drywall sample spectrum exhibits a cutoff shift to around 0.75 THz. The peak and cutoff frequencies reduce as the water content increases. The overall drop in power as water content increases further illustrates the THz wave’s high sensitivity to moisture.

### 2.4. Data Processing and Material Parameter Extraction

To extract the dielectric properties of the drywall at varying moisture levels, the time-domain waveforms acquired using the THz-TDS system were first transformed into the frequency domain using the Fast Fourier Transform (FFT). The reference and sample signals were processed to obtain amplitude and phase spectra.

Let ${\bar{E}}_{i}$ represent the incident reference THz field without passing through the drywall sample, and ${\bar{E}}_{t}$ the transmitted field passing through the drywall sample. Considering the relatively low contrast in refraction index between the drywall sample and background, a single-pass approximation was adopted in which the transmitted signal ${\bar{E}}_{t}$ is treated as arising solely from the first transmitted pulse. This approach assumes that reflections and transmissions occur only once at the front and back surfaces of the sample. As a result, multiple internal reflections between interfaces commonly modeled via Febry–Pérot interference analysis are ignored as justified by the small reflection at the boundary and the higher-order nature of the multiple internal reflections. The drywall is modeled as a lossy dielectric of thickness d, and complex refractive index $\bar{n}=n+j\kappa$.

In the frequency domain, the complex representations of the fields are given as:

(2) $${\bar{E}}_{i}=\left|E_{ref}\right|e^{j\emptyset_{ref}}$$

(3) $${\bar{E}}_{t}=\left|E_{samp}\right|e^{j\emptyset_{samp}}$$

By comparing the reference and sample fields, the ratio of the transmitted to the incident field becomes:

(4) $$\frac{{\bar{E}}_{t}}{{\bar{E}}_{i}}=\frac{\left|E_{samp}\right|}{\left|E_{ref}\right|}e^{j(\emptyset_{samp}-\emptyset_{ref})}$$

The real part of the refractive index n(f) is calculated using the phase difference between the sample and the reference [21,22]:

(5) $$n\left(f\right)=1+\frac{c}{2\pi fd}(\emptyset_{samp}-\emptyset_{ref})$$

The absorption coefficient α(f) is derived from the amplitude ratio using Beer–Lambert-like formulation [21,22]:

(6) $$\alpha \left(f\right)=-\frac{2}{d}\ln\left(\frac{\left|E_{samp}\right|}{\left|E_{ref}\right|}\cdot \frac{{\left(1+n\right)}^{2}}{4n}\right)$$

From α(f), the extinction coefficient κ(f) is computed as:

(7) $$\kappa \left(f\right)=\frac{\alpha c}{4\pi f}$$

The real part of the permittivity $\varepsilon^{\prime }$ is computed using:

(8) $$\varepsilon^{\prime }=n^{2}-\kappa^{2}$$

The imaginary part of the permittivity $\varepsilon^{\prime \prime }$ is given by:

(9) $$\varepsilon^{\prime \prime }=2n\kappa$$

The loss tangent tanδ is given by:

(10) $$\tan\delta =\frac{\varepsilon^{\prime \prime }}{\varepsilon^{\prime }}$$

All these computations were implemented in MATLAB R2022b using the experimental data collected from the six drywall states corresponding to 0%, 3.85%, 9.09%, 12.28%, 18.03%, and 21.88%. The correct phase difference was calculated using the phase unwrapping as demonstrated in [23]. The extracted material properties were plotted over the 0.2–0.65 THz range to examine the frequency-dependent dielectric behavior across moisture levels.

## 3. Results and Discussion

### 3.1. Refractive Index (n)

**Figure 5:**
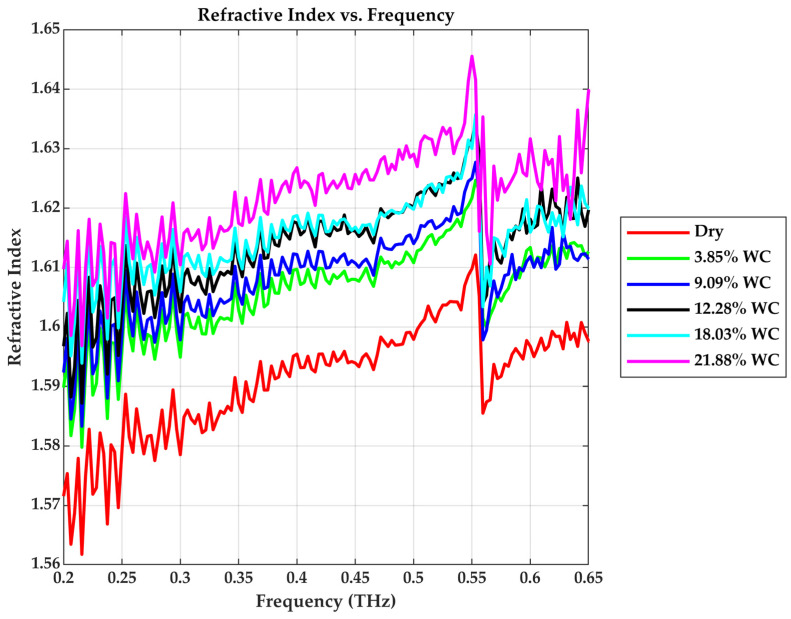
Refractive index vs. Frequency.

As shown in Figure 5, the real part of the refractive index n rises with the rising water content across the measured frequency range. For example, at 0.4 THz, the dry sample has a refractive index of approximately 1.595, while the moist samples progressively increased to 1.609, 1.612, 1.618, 1.619, and 1.627, indicating a clear dielectric response to moisture. The increase in the refractive index implies a reduction in THz phase velocity through wetter samples, resulting in longer optical path delays [24]. The sudden jump of the refractive index at 0.55 THz is due to water vapor absorption in air at this frequency [25].

### 3.2. Absorption Coefficient (α) and Penetration Depth (δ)

**Figure 6:**
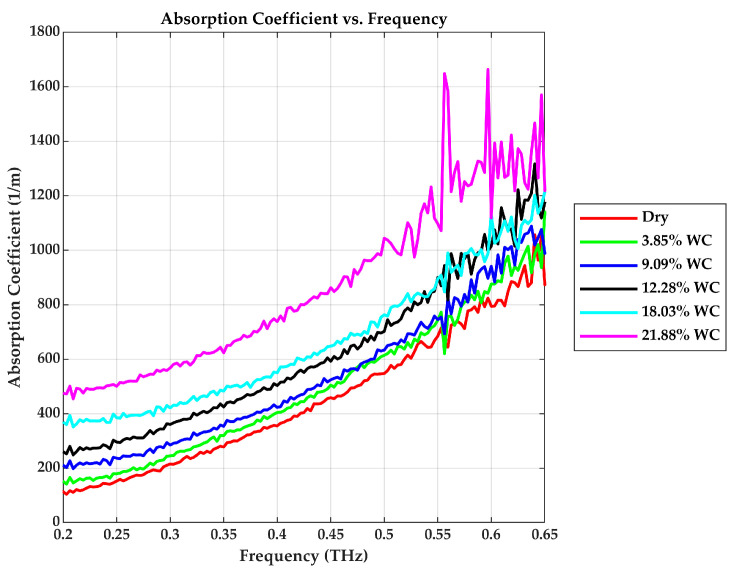
Absorption Coefficient vs. Frequency.

The absorption coefficient rises significantly with both frequency and water content. For instance, as plotted in Figure 6, at 0.5 THz, α increases progressively from approximately 570 $m^{-1}$ in its dry state to about 1070 $m^{-1}$ for the 21.88% *WC* sample, indicating more than an 87% rise. This confirms that water is a strong THz absorber, primarily due to its rotational and vibrational modes of the hydrogen-bonded water molecules that resonate at this frequency regime [26].

The exponential attenuation of the transmitted field follows Beer–Lambert’s law [17]:

(11) $${\bar{E}}_{t}\propto e^{-\alpha d/2}$$

**Figure 7:**
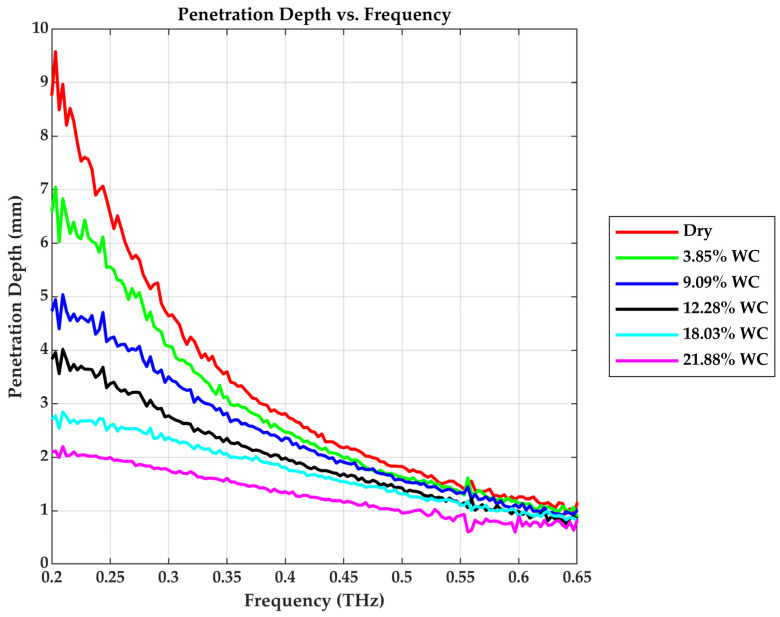
Penetration depth vs. Frequency.

This explains the significant amplitude decay in the frequency domain responses of moist samples, as shown in Figure 6. The attenuation depth (penetration depth) quantifies the extent to which the THz wave can probe into the material before significant absorption, and it is the inverse of the absorption coefficient. The penetration depth reduces with increasing water content in the drywall sample, as shown in Figure 7.

### 3.3. Extinction Coefficient (κ)

**Figure 8:**
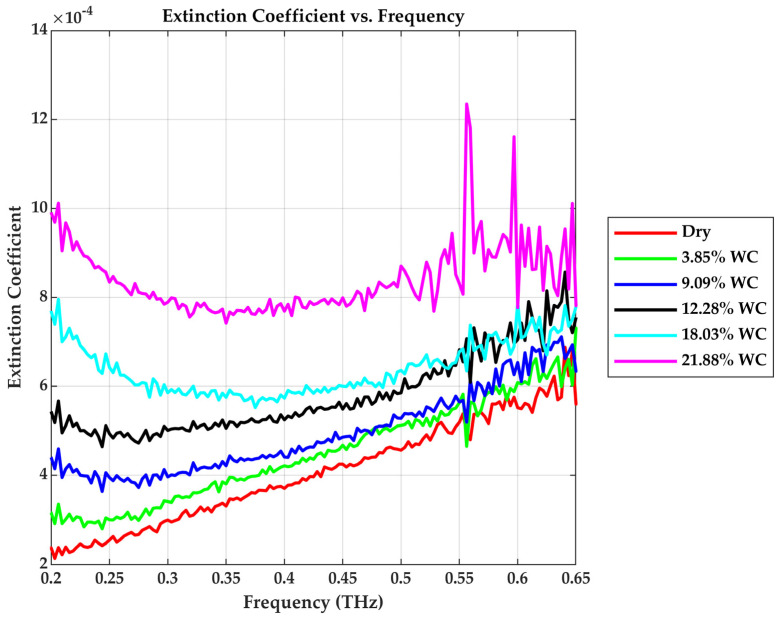
Extinction coefficient vs. Frequency.

The extinction coefficient represents the imaginary component of the complex refractive index. The extinction coefficient quantifies how much the amplitude of an electromagnetic wave decays as it propagates through a material due to energy loss from absorption, internal damping mechanisms, and scattering. As the THz wave enters the drywall sample, part of the electric field is dampened exponentially; the decay is due to the absorption by polar water molecules and any structural scattering inside the drywall material. The higher the water content, the larger the number of dipole oscillators, and the stronger the damping (extinction) of the wave, as evident in Figure 8.

The extinction coefficient is connected to the absorption coefficient with Equation (7). Although κ generally follows the trend of α, deviations at lower frequencies are observed due to its inverse frequency dependence. As frequency approaches 0.2 THz, the denominator is large and κ declines and flattens despite α increasing. The spectrum becomes more aligned with α at higher frequencies, where the frequency term in the denominator stabilizes.

### 3.4. Relative Permittivity ($\varepsilon^{\prime }$)

**Figure 9:**
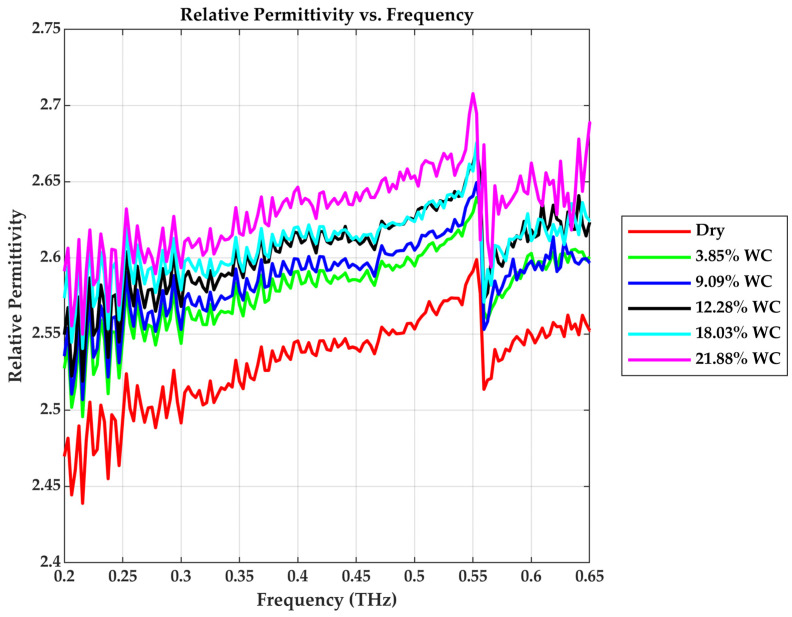
Relative Permittivity vs. Frequency.

The real part of permittivity ($\varepsilon^{\prime })$ reflects the drywall’s ability to store energy under an applied field and is directly related to the square of the refractive index. As shown in Figure 9, the permittivity of the dry Gypsum drywall varies slightly around 2.5 to 2.55 across the 0.2–0.65 THz. Increasing the water content enhances $\varepsilon^{\prime }$ due to the high dielectric constant of water molecules. For example, at 0.4 THz, $\varepsilon^{\prime }\;$ rises from 2.54 (dry) to 2.59, 2.60, 2.61, 2.62, and 2.65 for water levels of 3.85%, 9.09%, 12.28%, 18.03%, and 21.88%, respectively. The gradual increase in $\varepsilon^{\prime }$ signifies enhanced dipolar polarization under THz excitation as moisture rises. This is relevant in building diagnostics, as the dielectric constant is often linked to moisture in non-destructive testing [27].

### 3.5. Dissipation Factor (tan δ)

**Figure 10:**
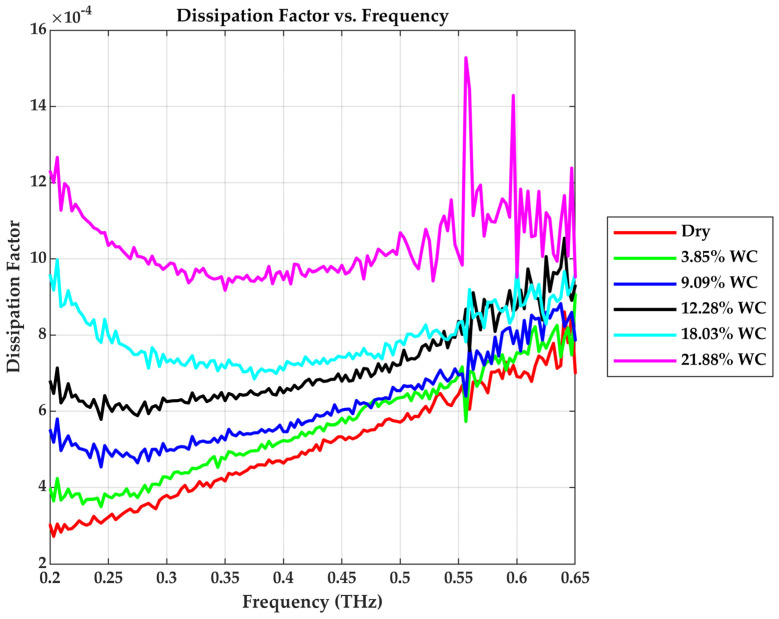
Dissipation factor vs. Frequency.

Dissipation factor (tan δ), also known as loss tangent, quantifies the ratio of energy lost to energy stored per cycle in a dielectric material. As shown in Figure 10, increasing moisture causes the loss tangent to increase, indicating that more electromagnetic energy is being converted to heat. For the frequency range considered, the dry sample exhibits a loss tangent value between $3\times 10^{-4}$ and $7\times 10^{-4}$ while with added moisture, the loss tangent can increase up to the order of $10^{-3}$ based on the amount of water content.

The trend highlights the dominant contribution of bound water loss mechanisms such as orientational polarization and dipole relaxations in the THz region. As water concentration rises, more bound water is present, leading to higher dielectric relaxation and energy dissipation [1]. Table 1 summarizes the dielectric properties at 0.4 THz.

## 4. Conclusions

This study investigated the dielectric properties of gypsum drywall at different moisture levels (0%, 3.85%, 9.09%, 12.28%, 18.03%, and 21.88%) using Terahertz Time-Domain Spectroscopy (THz-TDS). By extracting frequency-dependent material properties such as refractive index, absorption coefficient, extinction coefficient, relative permittivity, and dissipation factor, the quantitative analysis revealed that moisture content alters the response of drywall to THz pulses in the 0.2–0.65 THz range. These dielectric properties could be used to facilitate non-destructive moisture level detection within drywalls.

While the increase in the refractive index and relative permittivity with moisture content was attributed to the influence of water’s high dielectric constant and dipolar polarization effects, the interaction of THz waves with the vibrational and rotational modes of water molecules drives the increase in the absorption and extinction coefficients with increasing water content. The dielectric damping within the drywall sample accounts for the increase in dissipation factor with moisture. At intermediate moisture content levels, such as 12.28% and 18.03%, the transition from bound to free water molecules, influenced by thermal desorption dynamics, may introduce an additional non-linear absorption effect.

Beyond material characterization, the experimental results have important implications for THz propagation in indoor environments where drywall is a dominant architectural element. High permittivity and attenuation at high moisture levels can degrade link quality and reduce signal penetration for future short-range communication systems operating in the sub-THz and low-THz bands, which are of growing interest for 6G and high-capacity indoor wireless networks. The difference in dielectric properties between the sample at different moisture content levels also lays a promising foundation for non-invasive THz imaging and sensing applications such as hidden water leak detection, construction quality assurance, and structural diagnostics.

This study not only validates THz-TDS as a powerful non-destructive tool for material moisture testing, the experimental results also inform the electromagnetics modeling of building interiors under varying humidity conditions, paving the way for both scientific research and practical deployment of THz technologies in communication, sensing, and structural monitoring.

## Figures and Tables

**Table 1:** Table of dielectric properties at 0.4 THz.

Water Content (%)	n	$\alpha (m^{-1})$	κ	$\varepsilon^{\prime }$	tan δ	Penetration Depth (mm)
0	1.595	355.4	0.00037	2.54	0.00046	2.81
3.85	1.609	404.5	0.00042	2.59	0.00052	2.47
9.09	1.612	423.2	0.00044	2.60	0.00054	2.36
12.28	1.618	503.6	0.00052	2.61	0.00065	1.99
18.03	1.619	550.5	0.00057	2.62	0.00071	1.82
21.88	1.627	738.4	0.00077	2.65	0.00095	1.35

## Data Availability

The data are contained in the article.
